# Building a sanitary vulnerability map from open source data in Argentina (2010-2018)

**DOI:** 10.1186/s12939-020-01292-3

**Published:** 2020-09-24

**Authors:** Germán Federico Rosati, Tomás Alberto Olego, H. Antonio Vazquez Brust

**Affiliations:** 1 National Council of Scientific and Technical Research, CONICET, Godoy Cruz 2290, Buenos Aires, C1425FQB Argentina; 2 Institute for High Social Studies, University of San Martín, Av. 25 de Mayo 1021, San Martín, B1650HMI Argentina; 3 Bunge & Born Foundation, 25 de Mayo 501 6∘ Piso, Buenos Aires, C1002ABK Argentina; 4 Faculty of Social Science, University of Buenos Aires, Santiago del Estero y Carlos Calvo, Buenos Aires, C1075AAU Argentina

**Keywords:** Sample, Article, Health, Vulnerability, Census, Public policy, Open data

## Abstract

**Background:**

Designing public health policies to target the needs of specific places requires highly granular data. When geographic health statistics from official sources are absent or lacking in spatial detail, Sanitary Vulnerability metrics derived from Census and other georeferenced public data can be used to identify areas in particular need of attention. With that aim, a Vulnerability Map was developed, identifying areas with a substantial deficit in its population health coverage. As a result a novel methodology for measuring Sanitary Vulnerability is presented, that can potentially be applied to different time periods or geographies.

**Methods:**

Census, official listings of public health facilities and crowdsourced georeferenced data are used. The Vulnerability Index is built using dimensionality reduction techniques such as Autoencoders and Non-parametric PCA.

**Main results:**

The high resolution map shows the geographical distribution of a Sanitary Vulnerability Index, produced using official and crowdsourced open data sources, overcoming the lack of official sources on health indicators at the local level.

**Conclusions:**

The Sanitary Vulnerability Map’s value as a tool for place specific policymaking was validated by using it to predict local health related metrics such as health coverage. Further lines of work contemplate using the Map to study the interaction between Sanitary Vulnerability and the prevalence of different diseases, and also applying its methodology in the context of other public services such as education, security, housing, etc.

## Background

The notion of Sanitary Vulnerability^1^ is related with health determinants. In other words, there are certain factors and variables that are strongly linked with the health status - in an broad fashion, in terms of biology, psychology and social aspects- of a person or population. These determinants can be of different types and if they are absent or weak, a state of vulnerability occurs.

We built an indicator that tries to identify places where high Sanitary Vulnerability prevails. That is, places where it is not possible to attain the minimum threshold in terms of access to health services. To do so, articulating an operative definition of Sanitary Vulnerability is an important first step that guides the later surveying and processing of available information. Our operative definition is based on empirical micro-data generated by National Bureaus of Statistics, plus OpenStreetMap, and open data repository with global coverage. These readily available sources make our methodology well suited for countries in the Global South as the ones in Latin-American Region. Alternative methods, such as expert surveys (e.g. the Delphi Method) or population surveys, require fieldwork and dedicated resources representing significant costs, in particular when rural or hard to access areas are involved. We apply this methodology in Argentina, a country with fragmented statistical data and highly unequal in terms of sub-national economic development, paired with a vast territory with dispersed population in rural areas.

The Map we built is highly disaggregated: its basic unit of analysis is the Census Block, the smallest statistical unit in Argentina for which Census tabulations are publicly available. In urban settings a Census Block can be as small as a single city block. In rural areas, where population density is low, Census Blocks are much larger and can cover areas of several square kilometers. In addition to the Map itself we also introduce a novel methodology for estimating Sanitary Vulnerability, intended to produce updated metrics whenever fresh demographic data becomes available.

The paper is structured as follows:

- In “Methods” section we discuss the operative definitions for the indicators shown in the Map, we report the sources of information, the procedures and techniques used to process and analyze the information and we explain the different analytical methods used for the estimation of the Sanitary Vulnerability Index
- “Results” section analyzes geographical distribution of Sanitary Vulnerability Index and performs a validation
- In “Discussion and conclusions” we discuss limitations and further avenues of research.

### Definition of sanitary vulnerability

There are well known determinants linked to health status, related to unequal access to health services, both in terms of quantity and quality. The development of an index such as the one proposed here, aims at quantifying these inequalities to complement and contextualize other sources of health related information. It also raises awareness of particularly vulnerable regions. The estimation of a Sanitary Vulnerability could help identify and eventually prioritize “hot spots” on places with poor access to sanitary services that demand focused public health policy.

The presence of inequalities in the access to health services has been thoroughly studied and documented. Not all population strata show the same chances of accessing medical treatment. In general, the most vulnerable sectors and/or residents of low population density areas have a disadvantage in terms of access to health services [1, 2].

In specialized literature there is a differentiation between the notion of “inequality in access” with the notion of “vulnerability”, which is linked to a heightened risk to develop certain illnesses, or being exposed to certain factors of environmental risk.

An obstacle when trying to quantify “vulnerability” comes from the necessity to consider multiple factors that could explain inequalities in the access to the health system. In [3] the main dimensions and indicators linked to notions of vulnerability and inequality related to health care access are summarized.

Table 1 shows the main indicators from a number of previous studies analyzed in [3] such as those linked to experiencing poverty, belonging to a minority, suffering from chronic mental or physical illnesses, and lacking medical insurance. While these indicators are defined at the individual level, there are other factors affecting Sanitary Vulnerability that are prominently correlated with the environmental realm [4].

**Table 1:** Sanitary Vulnerability Indicators from [3]

**Vulnerability indicators**	**Papers**	**Percentage**
Poverty	21	91.3%
Ethnic or Racial minority	18	78.3%
Chronic physical or mental illnesses	12	52.2%
Lack of Health Insurance	8	34.8%
Old age	6	26.0%
Incarceration	3	13.0%
Migration	3	13.0%
Low level of education	3	13.0%
Residence in underserved areas	2	8.7%
Unemployment	1	4.3%
Widowhood	1	4.3%
Homelessness	1	4.3%

We evaluated many of the aforementioned indicators. Based on the availability of public data sets providing both nation-wide coverage and high spatial resolution, our approach for the Sanitary Vulnerability Index integrates two dimensions:

- Access to public -that is, universal, Government funded- health services: for this dimension, we used physical proximity to public health facilities^2^. A data set with the addresses of public health facilities throughout Argentina was compiled, based on data extracted from official listings published on websites ran by Municipal, Provincial and National authorities. These addresses were georeferenced to obtain their geographical coordinates. Then, for every Census Block in the country, a number of locations were sampled at random and then the shortest route to its nearest public health facility was calculated.
- Socioeconomic Status (SES): to construct a SES index we used Census information. As it will be detailed later, the indicator is based on Census 2010 data at the individual level. A series of relevant variables (education level, poverty indicators, etc.) were combined using variational autoencoders, a method for dimensionality reduction based on neuronal nets (see Section 5).

## Methods

The two dimensions mentioned before were combined into a georeferenced Sanitary Vulnerability Index that was projected onto the territory as a Sanitary Vulnerability Map. As discussed, the Map was designed to help identify areas with a potential deficit in sanitary services; that is, places were the local population access to health services lie below a minimum threshold. With this in mind, we built a metric to sort and classify the different zones in terms of their potential deficit.

### Data sources

To construct the Map, four different classes of sources were employed:

- Census data (National Census of Population and Homes and Households 2010 [5, 6])
- Georeferenced polygons representing Census Blocks.
- Names, service level and geocoded addresses of public health facilities: Public hospitals, health centers, and health posts (*n* = 16.564)
- Street grids (national and provincial routes, municipal roads) used to find the shortest path between households and health facilities.

All information was aggregated at the Census Block level, an areal unit.

In the following sections the procedures employed to process and analyze these diverse sources are explained.

### Distance to public hospitals and health centers

To build distance metrics, a unified, nation-wide health facility location data set was compiled by pooling different official data sources:

- National data set of Hospital and Primary Care Centers: based on the National Database of Hospital and Primary Care Centers (Base Nacional de Hospitales y Centros de Atención Primaria), which was compiled through the Sanitary Information System of Argentina (SISA) and published at the Argentine Republic Spatial Data Infrastructure (IDERA) site at http://catalogo.idera.gob.ar. This was considered the “master” data set, to be expanded with information from additional sources

Other health facility location sources:

- SUMAR health centers: SUMAR is a National program aimed at improving access to health coverage for people with no health insurance. Their official website lists thousands of public health facilities that were “scraped”^3^ and combined with those found in other sources.
- Hospitals and health care centers listed at the National Program for Sex Health and Responsible Reproduction website: Health facility lists, published as separate files for every Province, were downloaded and geocoded.
- Local government sources: information from several sources at the Provincial or Municipal level, usually from Provincial Ministries of Health websites, was also extracted and integrated in order to identify facilities not found in any of the National level sources.

Once the data was identified, the extraction, cleanup, geocoding and deduplication process was:

1. Data was downloaded either as a single consolidated file (when available) or iteratively scraped from its website.
2. Records with missing or unusable addresses were removed.
3. Health facility addresses were georeferenced using the Google Geocoding API.
4. All geocoded locations were overlayed
5. For each service-level category (hospitals, health centers, sanitary posts) geocoded facility locations were projected on a map, adding 100 meter buffer zone around each one. Where locations overlapped -i.e. their buffer areas intersected- those records were considered duplicates and only one kept. In that way, it was possible to identify and unify records obtained from different sources that used different addresses or names referring to a single site.

**Fig. 1:**
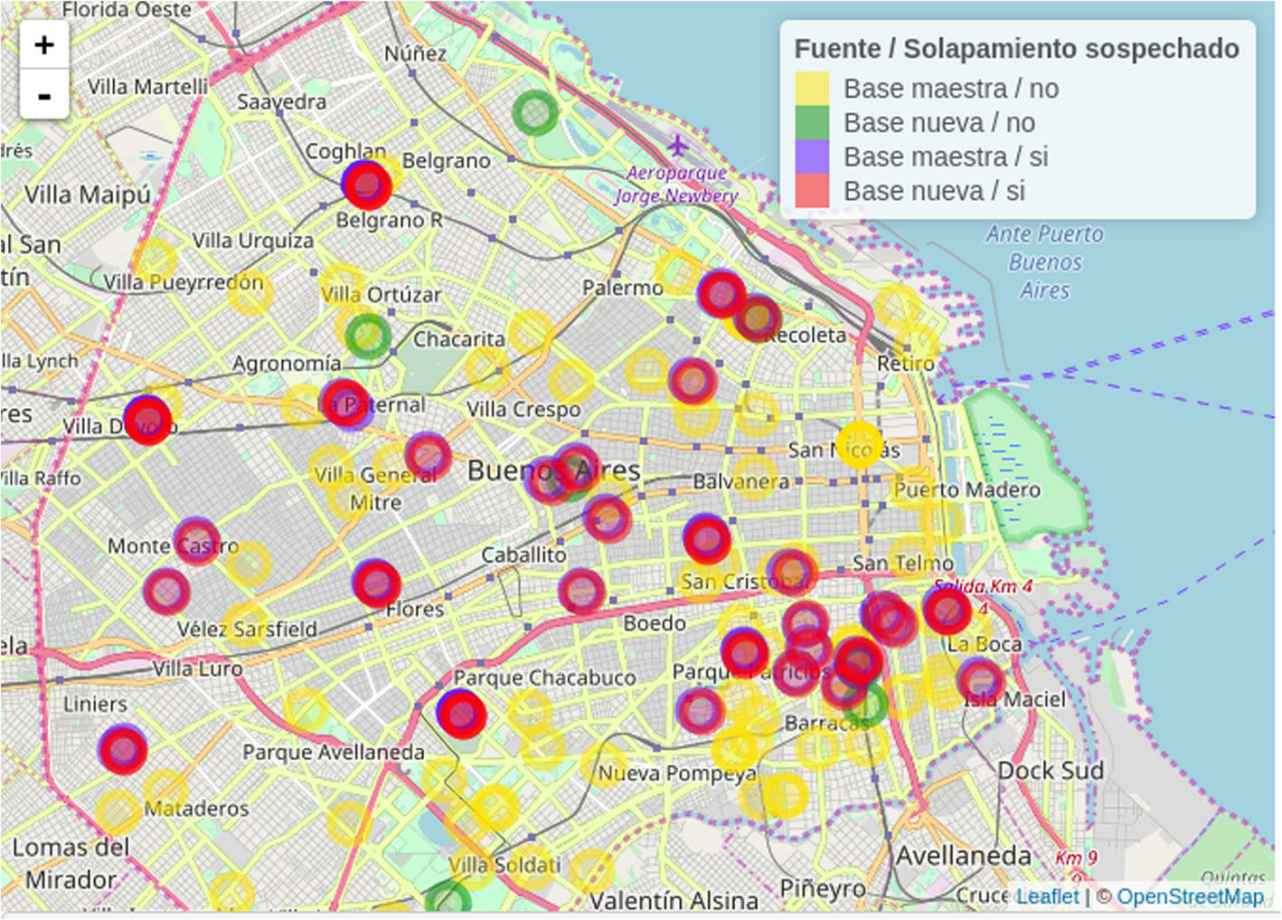
Comparison between master data set (SISA) and a municipal source. Buenos Aires City

As an example, health facility locations in Buenos Aires City, obtained from different sources, are shown in Fig. 1. The data set was clearly enriched by combining sources: while there are some overlapping sites corresponding to different data sets (red and blue dots), some others represent facilities that were found only on one of the sources (yellow and green dots).

#### Classification of health facilities according to their service level

After their location was established, public health facilities were classified according to their service level. Establishing their service level is important as not all health facilities are equal. Access to a hospital that offers specialized medical procedures improves the chances of receiving a better treatment, emergency care, and potentially better outcomes compared to being attended in a basic health services center (sometimes referred as a sanitary post in Argentina). As the data sources did not use an standardized system to indicate health service levels, an expert review was conducted with the help of a team of Argentine physicians experienced in public health administration and research^4^, who assessed the data and sorted the facilities into three categories, in decreasing order of health service capabilities:

1. Hospital
2. Health Center
3. Sanitary Post

These categories broadly reflect the complexity and variety of medical procedures available at each site. Hospitals provide the wider variety of services and regularly admit patients, including those requiring delicate surgery. Health Centers focus on outpatient services and sometimes a particular specialty, while Sanitary Posts aim to provide first aid and basic diagnosis.

After removing a number of facilities that do not belong to any of the categories (i.e. nursing homes, administrative offices, etc.), 15903 records were retained from the 16654 distinct places originally identified by merging disparate sources.

#### Travel time to the nearest public health facility

Physical accessibility has been recently used as a proxy to unequal distribution of goods and resources. A recent paper by [7] shows that there is a positive correlation between aggregated time access to urban agglomerates and several common socioeconomic indicators: income level, educational level, child mortality rates, etc.

In order to define an accessibility metric based on household proximity to Public health facilities, distance and travel time to the nearest facility was calculated for every Census Block in Argentina.

To solve the issue of the varied shapes and areas of Census Blocks -quite different across the country, with a stark contrast between high and low density areas-, distances and travel times to the nearest facility was calculated by sampling many locations at random inside each Block. The procedure is as follows:

1. Five points are randomly chosen for each Census Block
2. For each point, the nearest health facility is identified
3. For each point, travel time and distance traversing the local street grid to its nearest facility is calculated.
4. Distances and times are averaged.

The procedure was repeated for facilities in each of the service level categories (see previous section). To identify the nearest facility, a kNN algorithm -k nearest neighbors- algorithm was used to compare the coordinates of the samples locations and the facility addresses.

To find the optimal route between the two points we used Open Source Routing Machine (OSRM), a high performance routing system that can find the shortest path between locations through the public road network, by foot and by car [8]. Detailed road data was obtained from OpenStreetMap [9], an open, global, crowdsourced database whose quality has made it a frequently used source in mobility studies [10, 11].

#### Travel time on foot

As an accessibility measure, walking distance and time (travel on foot) was chosen over travel by car as there is evidence -at least for certain medical treatments- that the distance by foot to the health facility is a good predictor of treatment outcome.

Results from this study of Baltimore City clients attending outpatient drug addiction treatment programs suggest that having to travel more than 1 mile from the treatment center reduces medical patients’ chances of completing treatment by almost 50%, after controlling for the effects of demographic variables and type of drug problem. Moreover, living more than 4 miles away from treatment decreases the expected length of treatment by almost 13 days in comparison to clients traveling less than 1 mile [1].

Moreover, [1] indicates that distances longer than a mile (approximately 1.6 km) affect treatment outcome, highly increasing the chances of a patient abandoning rehabilitation treatment. When the distance exceeds 4 miles (6.4 km), the expect duration of a treatment increases by almost two weeks.

For a very reduced number of Census Blocks it was not possible to determine the travel path on foot to the nearest facility (10 out of 52406 Blocks analyzed). For each of them, visual inspection of aerial imagery indicated that they comprise either rural areas near lakes or estuaries, or large plots dedicated to agriculture, where no paths are present. In those instances, the largest distance found for neighboring Blocks was used instead.

**Fig. 2:**
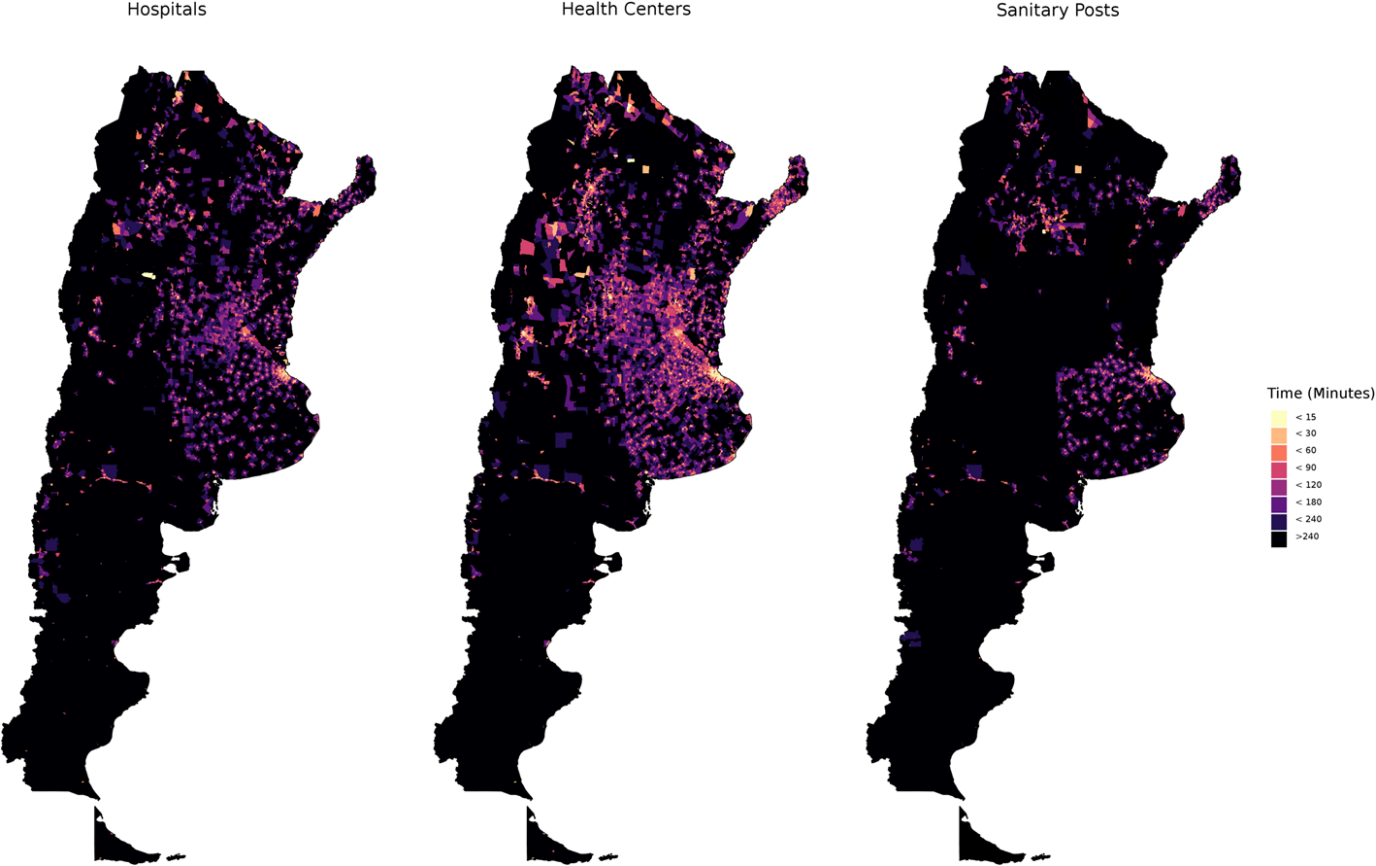
Travel time (on foot) to different classes of health facilities. Census Block Average

Figure 2 shows mean traveling times on foot to the nearest hospital, health centers and sanitary posts in Argentina.

The results for hospitals and health centers are quite similar. In contrast, the map of sanitary posts shows a clearly different pattern. There is a significant portion of the Country where travel times to the nearest sanitary post are very high. Closer inspection indicates that there exists an inverse relation between distance to hospitals / health centers and distance to sanitary posts.

This difference could be explained by two non mutually exclusive reasons:

- As local Governments decide the location (even the mere existence) of sanitary posts, the absence of sanitary posts could be explained by lack of local interest in setting up posts, or lack of means.
- Incomplete sources: that is, additional posts exist but are absent from official listings of health facilities.

Even with these caveats regarding the completeness of the data, travel distance to the nearest sanitary post was still included as part of the accessibility indicator. Since the indicator is built based it on a median function, the effect of potential outliers is minimized.

For each Census Block *Δ*_*r*_ is the median distance for all sampled locations inside it, to the nearest facility of each service level class: hospitals (Hosp), health centers (HCter) and sanitary posts (SPost):

1 $$ {}{\begin{aligned} \Delta_{r} &= MED(\Delta_{Hosp_{1}},..., \Delta_{Hosp_{5}}, \Delta_{HCter_{1}},..., \Delta_{HCter_{5}},\\ &\qquad\qquad\Delta_{SPost_{1}},...,\Delta_{SPost_{5}}) \end{aligned}}  $$

### Socioeconomic status -SES-

The second dimension of Sanitary Vulnerability represents a summarized measure of different SES characteristics of the population. This measure represents the population social and economic conditions, considered a key factor for the differences in sanitary risk across different populations.

Data from the 2010 National Census of 2010 at the individual level was available. Since SES is usually measured at household level, we calculated an index for each head of household in the data set from the Census.

Our SES index tries to ameliorate a key limitation in the most commonly used poverty index in Argentina, known as the Unmet Basic Needs index (*indicador de Necesidades Básicas Insatisfechas*). The Argentine Census provides this indicator, computed at the household level. Each household has only two possible states, “poor” or “not poor”, resulting from a set of binary indicators reflecting dwelling construction materials, household composition, etc. If any of these indicators fails to meet a threshold value, the household is considered poor. In contrast, our index models Socio-Economic level as a gradient. As an example, for the Unmet Basic Needs index the dwelling construction quality is dichotomic: either the household inhabits a deficitary dwelling, or it does not. Instead, our SES index computes this variable as a scale of four states. This approach allowed us to represent not only the poorest households at the bottom of the SES scale (as the Unmet Basic Needs index does) but also the members of the much smaller top section: households with very good living conditions.

To calculate the index the following variables (Table 2) were used, ordinally classified.

**Table 2:** Indicators used for SE Index

**Variable**	**Unit**
Dwelling property condition	Dwelling
Construction Material Quality	Dwelling
Basic Services Access Quality	Dwelling
Dwelling Construction Quality	Dwelling
Overcrowding	Household
Basic Necessity Index	Household
Head of Household Educational Level	Household
Number of Unemployed persons in Household	Household
Domestic Service Existence	Household
Employment Status	Individual (head)
Educational Level	Individual (head)

To construct the SES index a “thermometer encoding” for ordinal variables was used. Let *N* be the number of cases and *v*1,…,*vI* the variables. For each variable *v*_*i*_, there are *K*_*i*_ categories. We created the following codified variables $x^{(i)}_{k_{i}}$ for each variable *v*_*i*_ and for each *k*_*i*_ category where 2≤*k*_*i*_≤*K*_*i*_. For each case *j* with 1≤*j*≤*N* we have:

2 $$ x^{(i)}_{k_{i}}(j)= \left\{\begin{array}{ll} 0, & \ if \ v_{i}(j) < k_{i} \\ 1, & \ if \ v_{i}(j) \geq k_{i} \end{array}\right.  $$

For example, dwelling construction quality is and ordinal variable, based on the INMAT index^5^. INMAT values, ranging from 1 (lowest construction quality) to 4 (highest quality), were represented as shown in Table 3.

**Table 3:** Example of thermometer encoding

**INMAT original**	**INMAT_2**	**INMAT_3**	**INMAT_4**
1	0	0	0
2	1	0	0
3	1	1	0
4	1	1	1

In this example, *INMAT*_1_ would be the baseline or reference category.

#### Construction of a SES index

The final index was computed using an autoencoder, a technique of dimensionality reduction [12, 13], based on neuronal networks architecture. In short, an autoencoder has the objective to find a representation of input data (encoding) usually with the aim of reducing dimensionality. In terms of statistical and data-science practice, modern literature tends to favor the use of autoencoders because they can be applied to manifold learning, handling nonlinear dimensionality reduction. Autoencoders also offer better generalization properties as they implement regularization methods. As an additional incentive, our data set contains mostly ordinal and categorical data which tends to be problematic for other methods

An autoencoder has two elements:

- an encoder (or recognition network) that converts inputs to an internal representations, followed by a
- decoder (or generative network) that re-converts the internal representation to outputs.

It has an architecture analogous to a Multi-Layer Perceptron, but the number of neurons in the output layer has to be equal to the number of neurons of the input layer. The trained model has a logistic function as a final layer and regularizes using a dropout (of 0.5) in the intermediate layers to improve generalization performance.

**Fig. 3:**
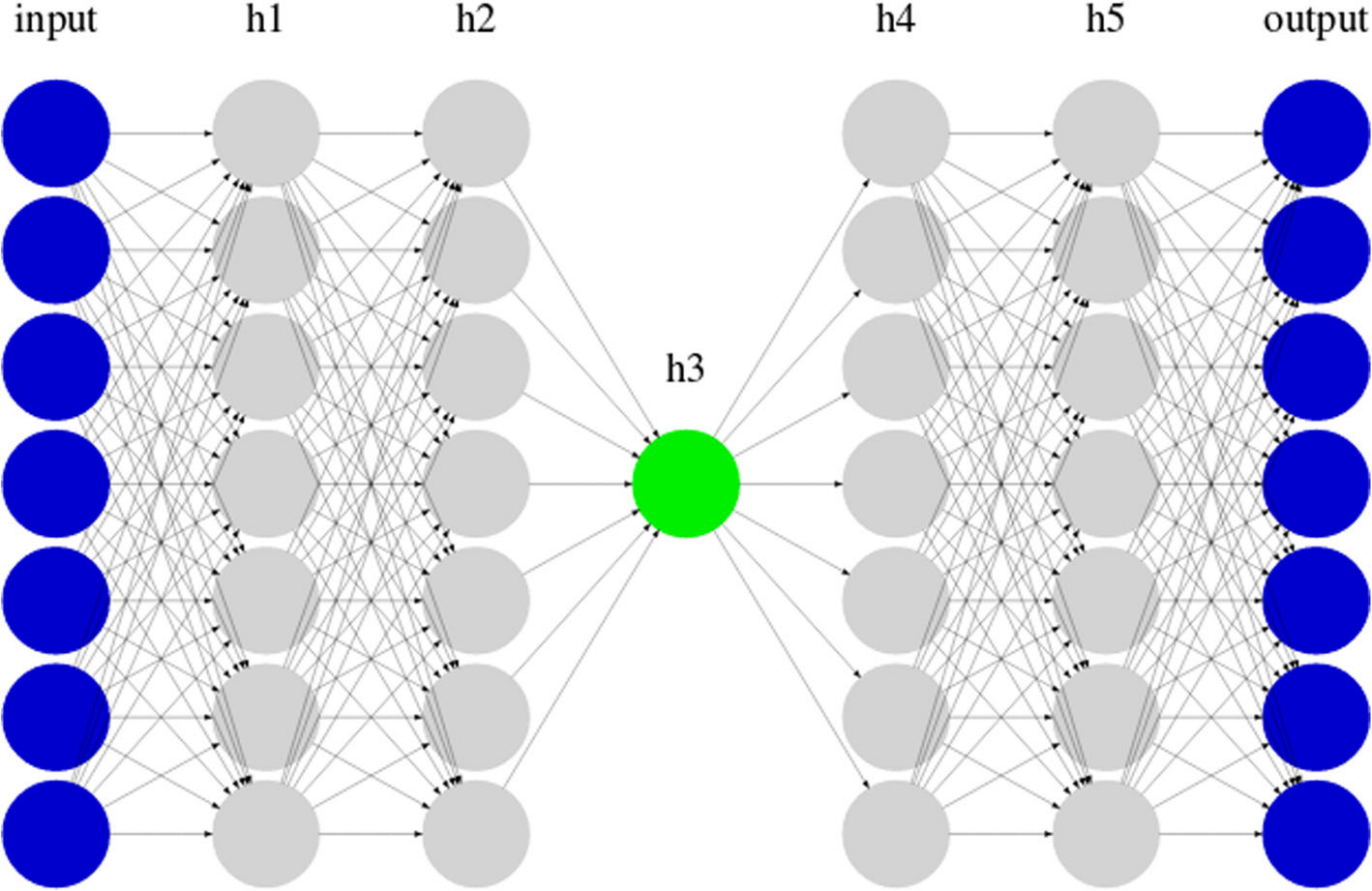
Autoencoder model used

The estimated model was the following (also graphically shown in Fig. 3):

$$\begin{array}{*{20}l} h_{1} &= \tanh (W_{1} (x * r_1) + b_{1}) \\ h_{2} &= \tanh (W_{2} (h_{1}* r_2) + b_{2}) \\ h_{3} &= \tanh (W_{3} (h_{2}* r_3) + b_{3}) \\ h_{4} &= \tanh (W_{4} (h_{3}* r_4) + b_{4}) \\ h_{5} &= \tanh (W_{5} (h_{4}* r_5) + b_{5}) \\ \hat{x} &= \sigma (W_{6} h_{5} + b_{6}) \end{array} $$

where *W*_*i*_ is the coefficient matrix of layer *i*, and *b*_*i*_ is the mean of layer *h*_*i*_. In this way, the log-likelihood for each case can be written as

$$\begin{aligned} \Pi(\hat{x}, x) = \sum_{i = 1}^{I} \sum_{k_{i} = 2}^{K_{i}} w^{(i)}_{k_{i}} \bigg(x^{(i)}_{k_{i}} \log \left(\hat{x}^{(i)}_{k_{i}} \right) + \left(1-x^{(i)}_{k_{i}} \right) \log \left(1-\hat{x}^{(i)}_{k_{i}} \right) \bigg) \end{aligned} $$

where $w^{(i)}_{k_{i}}$ is defined for each variable *v*_*i*_ with its *K*_*i*_ categories as:

$$\begin{array}{*{20}l} w^{(i)}_{k_{i}} &= \frac{1}{K_{i}} \cdot \left(\frac{ \sum_{\ell} K_{\ell} }{I} \right) \end{array} $$

.

The loss function was defined as a weighted likelihood:

$$\begin{array}{*{20}l} L(\hat{x}, x) = \text{argmin}_{W, b} & \sum_{j=1}^{N} \Pi(\hat{x}(j), x(j)) \end{array} $$

The model was trained with ADAM [13] defining batches of data resampled with repetition over the empirical distribution to ensure convergence.

Since the model include the population data (the data set is the Census itself), the aim was the generation of a descriptive measure. So, the model evaluation criteria was the weighted average of the probability of each variable-category:

$$\begin{aligned} & Error(\hat{x}, x) = \frac{1}{N} \sum_{j = 1}^{N} \sum_{i = 1}^{I} \sum_{k_{i} = 2}^{K_{i}} \frac{1}{\sum_{\ell} K_{\ell}} e^{x^{(i)}_{k_{i}} \log \left(\hat{x}^{(i)}_{k_{i}} \right) + \left(1-x^{(i)}_{k_{i}} \right) \log \left(1-\hat{x}^{(i)}_{k_{i}} \right) } \end{aligned} $$

The final model preserves 85% of the total information of the input.

In this way, from *h*_3_ on, each household head, and therefore, each household, is classified with a value resulting from the autoencoder, which will be called *s*_*i*_, SES.

Using SES, a socioeconomic indicator was created from an aggregated measure for each Census Block using data from the head of household head and it SES. So, for each household head *i* with SES *s*_*i*_ living in the Census Block *r* with a population of *n*_*r*_ head of households *η* as

3 $$ {}\eta_{r} = \frac{1}{4} Q_{.25}(\mathbf{s}_{r_{1,...,n_{r}}}) + \frac{1}{2} Q_{.5}(\mathbf{s}_{r_{1,..,n_{r}}}) + \frac{1}{4} Q_{.75}(\mathbf{s}_{r_{1,..,n_{r}}})  $$

where *Q*_*p*_ is the *p* quartile and *η*_*r*_ the result of applying the summary measure known as “Tukey Trimean”, which delivers a compromise between robustness and efficiency, compared to the mean.

**Fig. 4:**
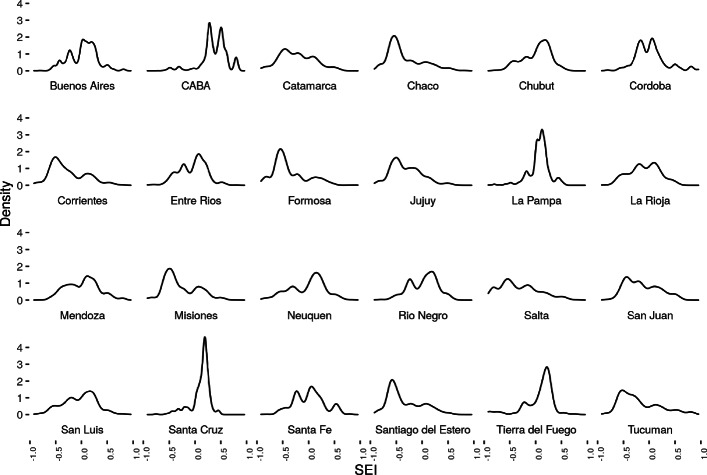
SSE by Province density plot
- Provinces such as Chaco, Formosa, Jujuy, Salta, show left skewed distributions (lower SES index).

Figure 4 shows that the index seems to capture well known disparities between Provinces:

- The city of Buenos Aires presents a distribution clearly skewed to the right (higher values of SES index)

### Estimation of a sanitary vulnerability index

The index of Sanitary Vulnerability design took into consideration the following dimensions:

- Socioeconomic Level
- Access to health center

As said, Socioeconomic level can be considered a determinant of the population situation related to sanitation and social resources (such as education, and to conditions of overcrowding and habitability of the dwelling). Some of these resources are related individual and social trajectories and histories. Nevertheless they also have a strong relationship with residence place. In other words, the Socio Economic Level is linked to the distribution problem of and therefore it is an intensive variable.

To construct the index of Sanitary Vulnerability it is necessary to combine both variables at Census Block level. Principal Components Analysis (PCA) is well-suited for this: we are looking to estimate a linear combination between two variables, each one with different assumptions and statistically independent, so a simple average will not suffice. Therefore, we will use a non-parametric PCA in order to combine them while avoiding any statistical assumptions, searching for a linear combination of the variables that maximizes explained variance.

When the variables show atypical distributions, for example multimodal, asymmetric or fat-tailed, PCA interpretation can be difficult: the method is sensitive to the variable scaling [14]. One possible solution is to transform variables using ranges of data [15, 16].

Therefore, following [17], each *X*_*j*_ was standardized using a rankit transformation:

4 $$ rankit(X_{i,j}) = \frac{r_{j}(x_{i,j}) - 0.5}{n}  $$

For each observation i of each variable *X*_*j*_ the rank is calculated *r*_*j*_(*X*_*i*,*j*_) -ranging from 1 and *n* -, 0.5 units are subtracted and it is divided by the total number of registries *n*.

As a result, differences in units between variables, changes in scale, displacements and monotone transformations are removed.

**Fig. 5:**
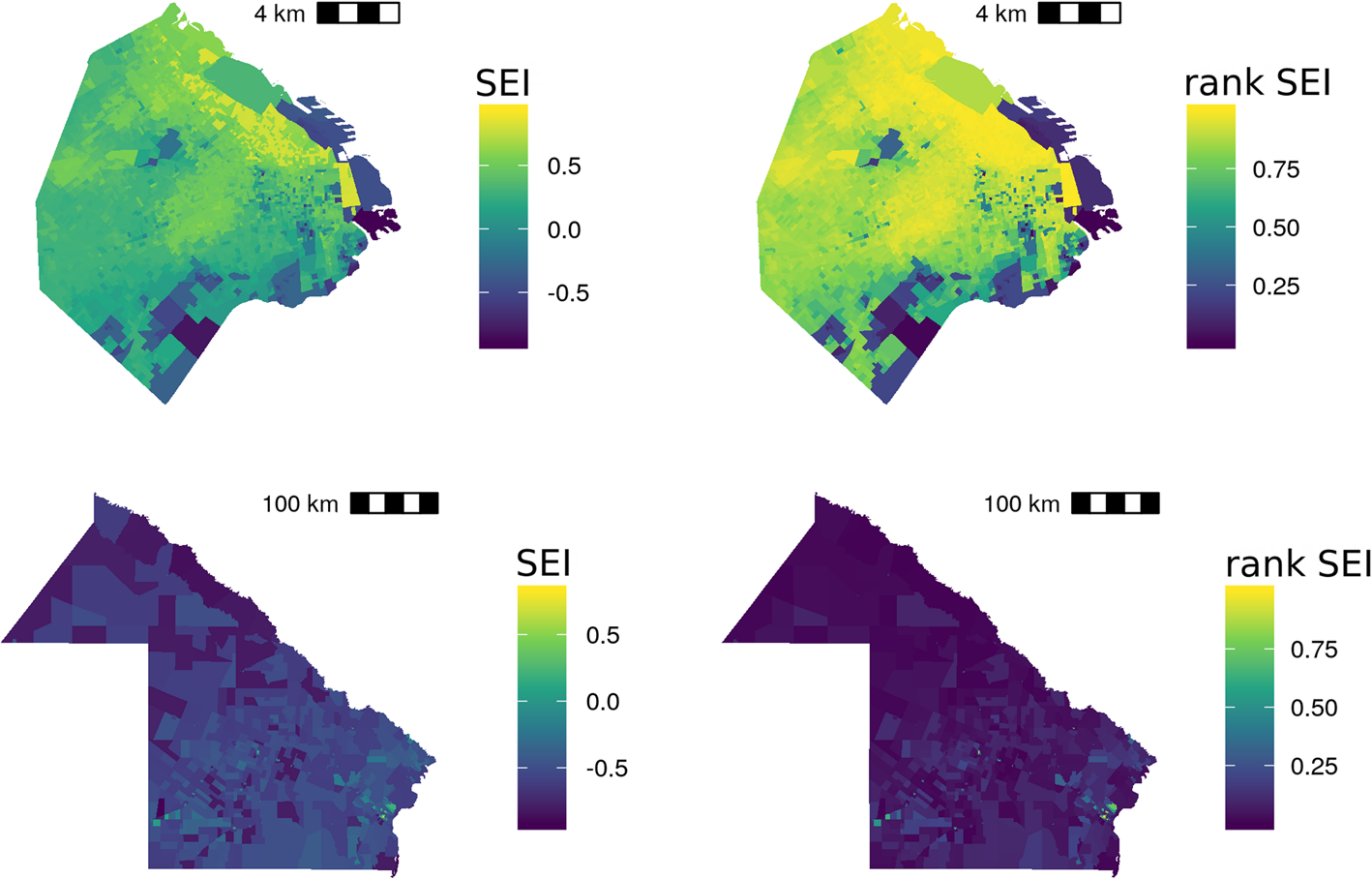
SES without and after the rankit transformation. City of Buenos Aires and Chaco Province

**Fig. 6:**
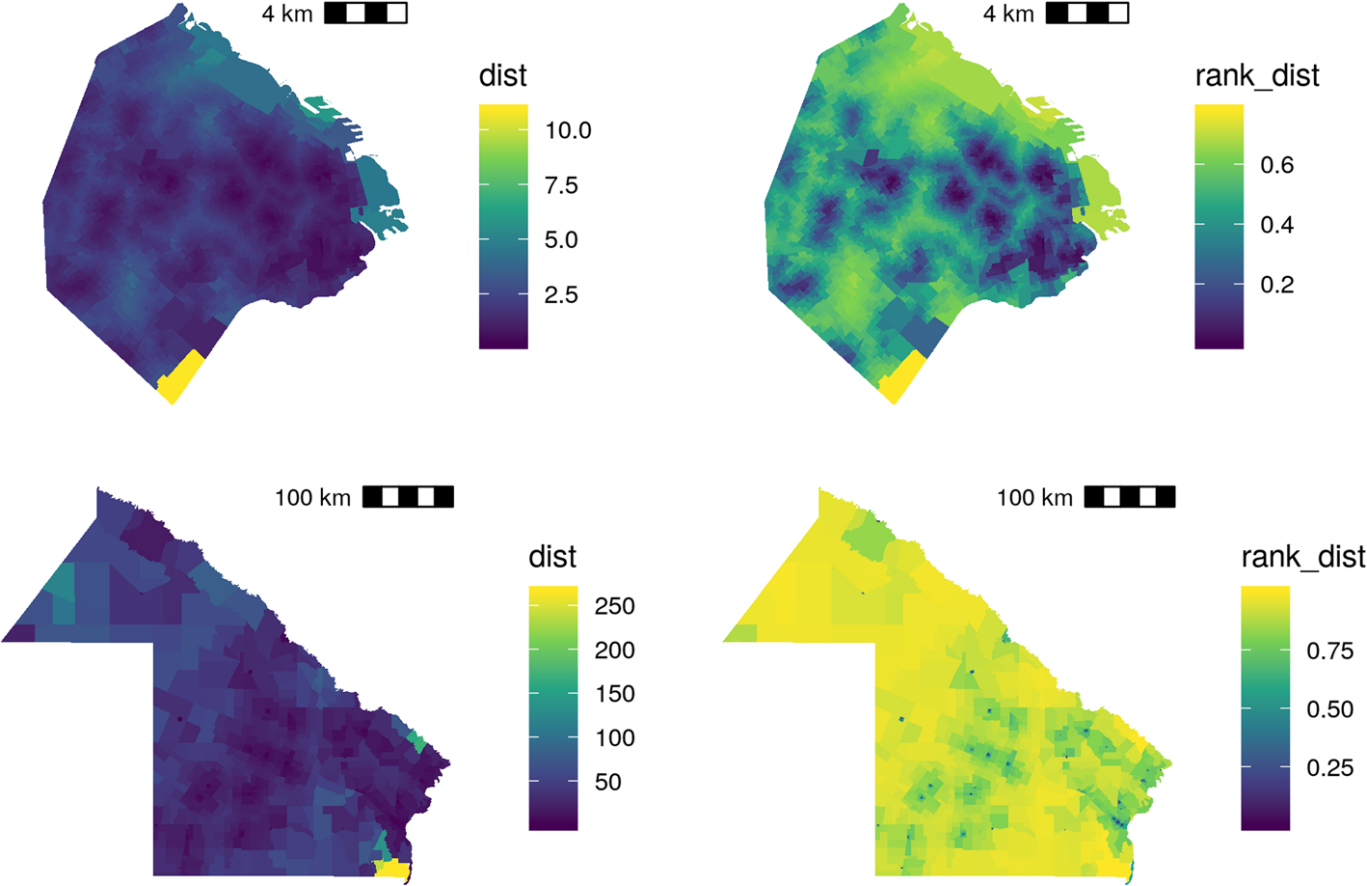
Distances to health centers, before and after the rankit transformation. CABA and Chaco Province

Figures 5 and 6 show that the rankit transformation effect in SES - *η*_*r*_- and access to health units - *Δ*_*r*_-.

It is easy to note that rankit is simply a non parametric estimator of the c.d.f. Following Egger et al. [18] y a Han y Liu [19], a Semiparametric Principal Component Analysis was performed, as shown in Algorithm 1.


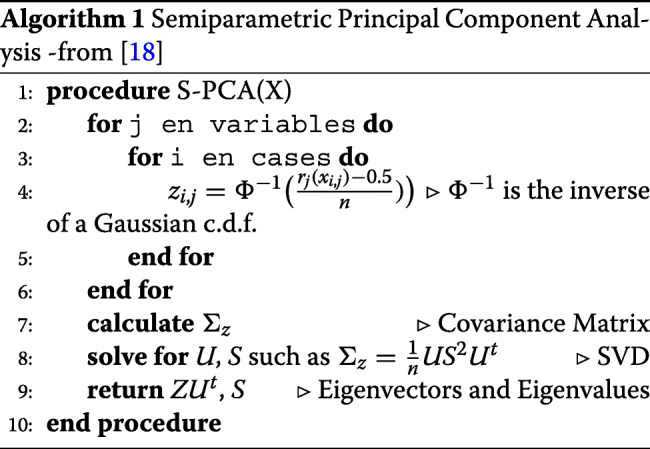


To combine both variables, it was necessary to calculate Spearman’s correlation between rankits [19]. Then the non-parametric correlation matrix was decomposed. The first autovector absorbed 72% of the variability and was oriented in the opposite direction of mutual growth. This first autovector was considered the optimal combination between both variables. And after successive application of non parametric transformations, and index tolerant of contamination and above all independent to the scaling characteristics of each entry variable.

The aim was to obtain an index between 0 and 1 whose distribution were homogeneous for all units. There was a new application of a c.d.f - type estimated through log-splines using AIC as criterion of regularization over the main direction, deriving in this way the index *VS*_*r*_ for each Block.

In difference to rankit, when modeling the distribution with log-spline, relative distance are respected.

**Fig. 7:**
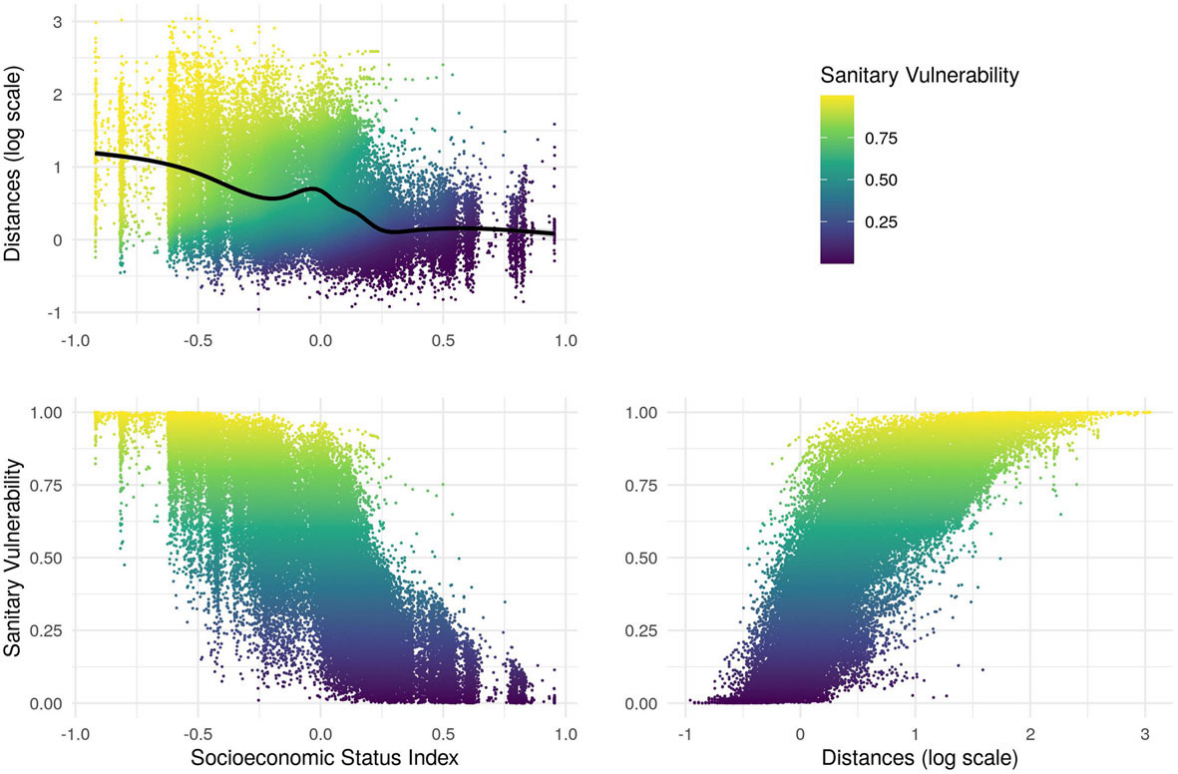
Sanitary Vulnerability Index

In the first graph of Fig. 7, result of S-PCA is shown in the space of Z, and the colors show the value of the resulting index. In the second graph, the result is observed in the space of X. Finally in the to graphs below of Fig. 7, the index is shown as a function of SES index and distances, respectively.

## Results

**Fig. 8:**
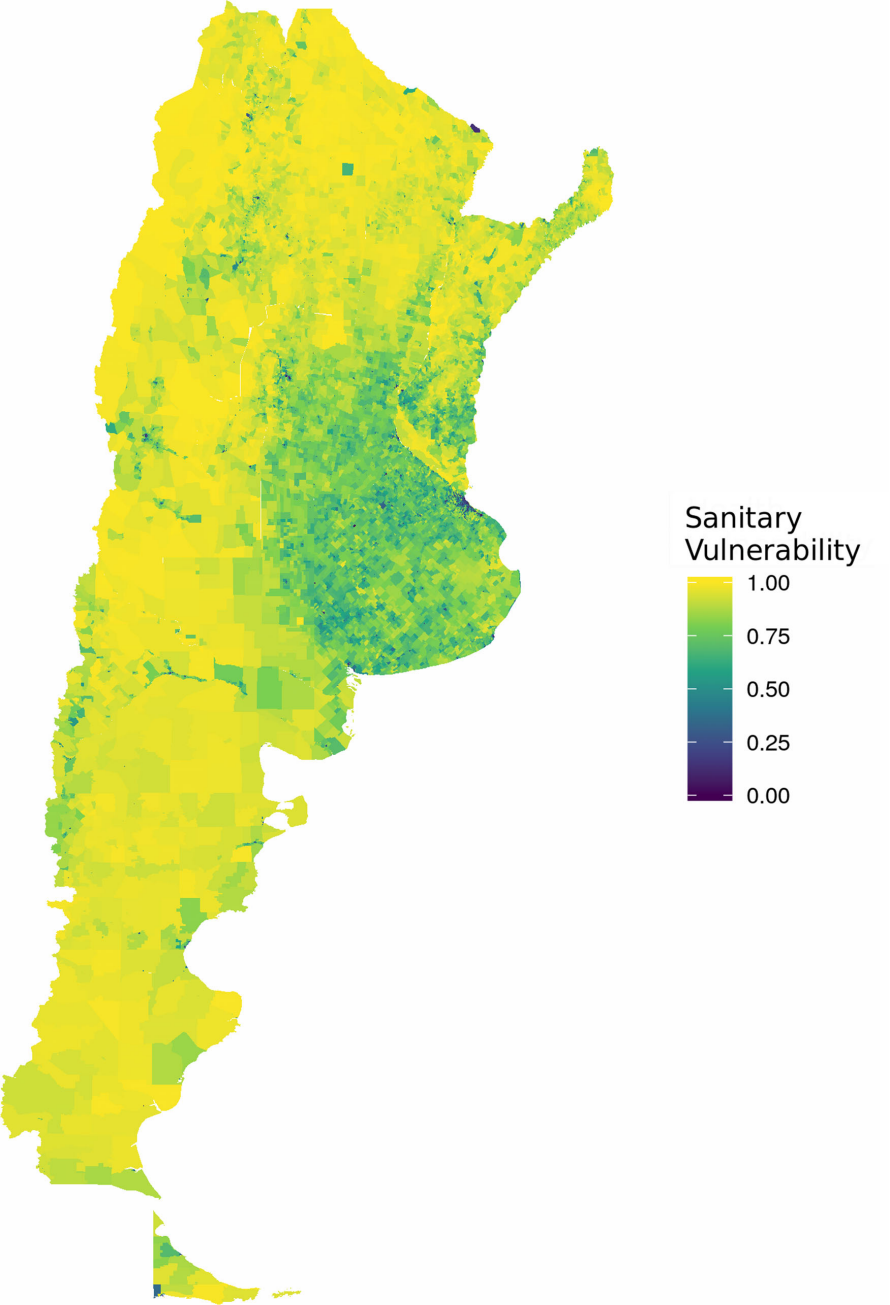
Sanitary Vulnerability Index. Argentina

Figure 8 shows a map with the Vulnerability index at the census tract level.

Now, this visualization needs to be taken with care due to the scale employed. In fact, the previous map seems to represent an expected situation where two zones can be delimited:

- “Central” Region: Province of Buenos Aires, City of Buenos Aires, and the large urban agglomerates of each Province, characterized by higher values of Sanitary Vulnerability.
- The rest of the country, mainly in less populated regions, with critical values.

When aggregating information one level higher (census fraction) the result is slightly different.

**Fig. 9:**
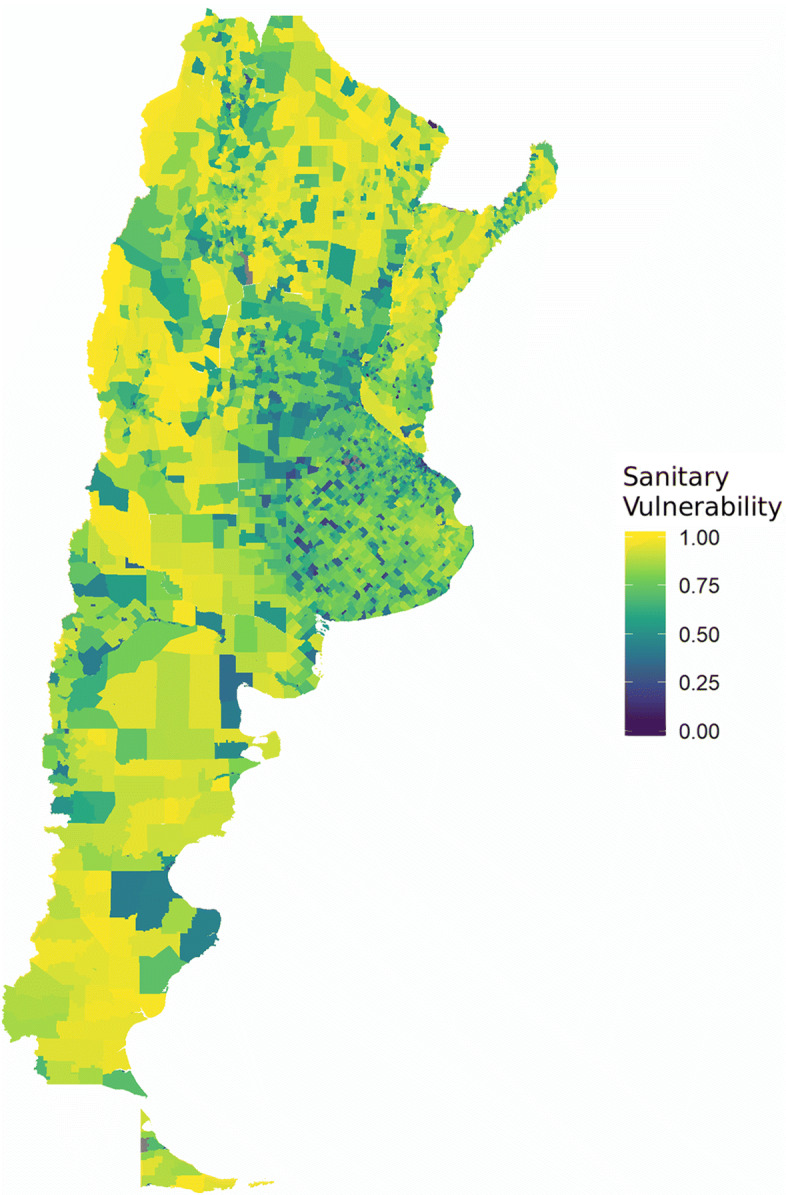
Sanitary Vulnerability Index at Census Fraction level. Argentina

Figure 9 shows a mixed version: the regions previously detected are not so stark. They are observed in the central zone with critic values. In the non-central zone, there are better ranked sectors.

**Fig. 10:**
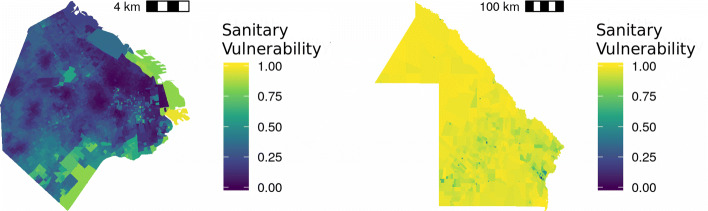
Index of Sanitary Vulnerability:. CABA (left.), Chaco (right)

Spotting certain areas (but keeping the level of disaggregation by Census Block), there are patterns that are invisible in the aggregated map. If the aggregated map is displayed for the Provinces of Chaco and CABA the following result arises (Fig. 10).

Then, in CABA, it is possible to detect higher Sanitary Vulnerability pockets, and in Chaco there are places presenting satisfactory values for this indicator.

### Validating the sanitary vulnerability index

This section will attempt a first validation of the Sanitary Vulnerability Index (SVI) by complementing it with other statistical sources.

If the SVI is valid, it should be safe to assume that higher proportions of population without medical coverage are associated with higher SVI values. In fact, the medical coverage of the population, that is, the access they have to the health system should show a negative correlation with the index values.

In this regard, it is necessary to clarify one point: the variable linked to the type of medical coverage of the population was not used for the SVI because the Population Census did not include a question for it in its basic questionnaire (which allows disaggregation at the Census Block level), only in the expanded one^6^. The latter allows for a disaggregation only at the Department (an aggregation level comparable to the County level in the United States) level. That is why we will compare both distributions (SVI and health coverage) at the Department level.

This exercise will have the following characteristics:

1. it will be based on census radio information
2. different aggregate measures will be calculated at the department level
3. it will be compared with the proportion of the population with medical coverage (that is, prepaid or social work) at the departmental level

**Fig. 11:**
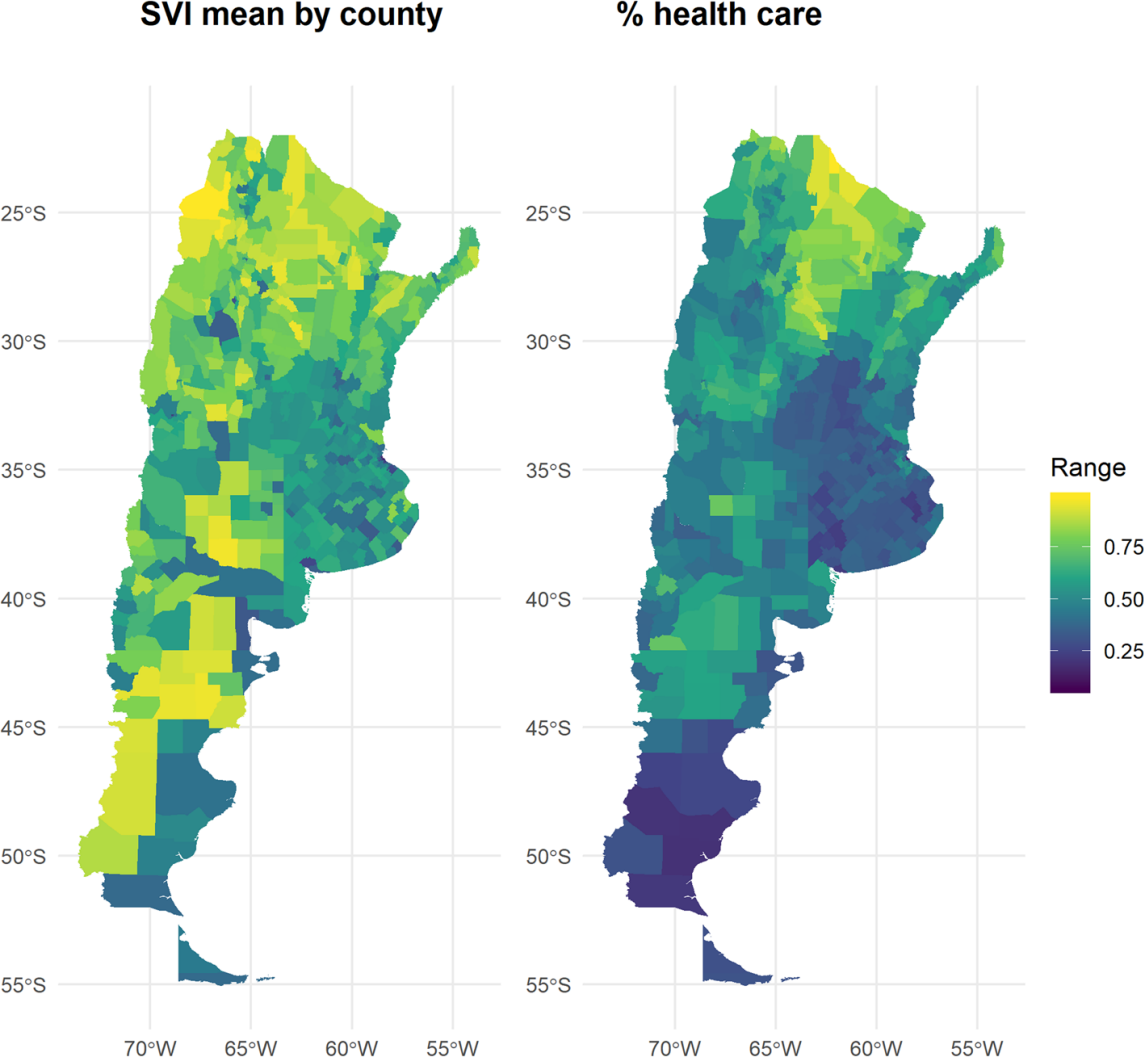
Sanitary Vulnerability Index, by population proportion with no health care coverage (county level)

**Fig. 12:**
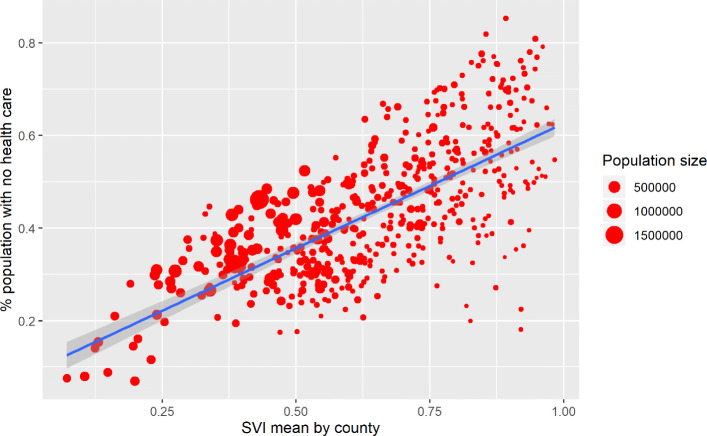
Sanitary Vulnerability Index, by population proportion with no health care (county level)

The following plots show the geographical distribution of both variables (Fig. 11) and a scatterplot (Fig. 12).

When analyzing the correlation between both variables, it is observed that at higher average values of the SVI, greater proportions of medical coverage in the population are observed: Pearson’s R correlation is about 0.70.

However, it can be seen that the departments with higher values of the SVI, correspond to a greater dispersion in the non-medical coverage of the population. In turn, these departments are the smallest in terms of population size.

## Discussion and conclusions

Having validated the Sanitary Vulnerability Map’s capacity to predict local health coverage, it seems to be a valuable tool for policymaking, as it provides a spatially granular estimate of the population’s access to public health services.

Its nation-wide coverage and geographic disaggregation also makes it a valuable resource for the study of public health issues in Argentina. A recent analysis of health access barriers based on two national surveys found that neither had reached a population diverse enough to make conclusions about socioeconomic differences in access levels [20]. As the SV index has been estimated for the entire territory, covering the gamut of socioeconomic settings, it can be used to contrast and supplement the results of surveys.

Researchers monitoring access to public health services also found a frustrating absence of "publicly available [geographically] disaggregated indices" from Argentina’s National Ministry of Public Health, thus being limited to evaluate metrics summarised at the national or regional level [21]. The SV index, publicly available at Census tract level, contributes by providing a highly disaggregated metric, including a novel sub-component representing physical distance between homes and public health care providers. In that respect, both the method for collecting and georeferencing public health facilities, and the database that resulted from the process also constitute an important resource for future research on public health coverage; i.e. by allowing to use GIS techniques to estimate catchment areas, find optimal placement for new facilities, etc.

On the other hand, a current limitation of the Sanitary Vulnerability Index is that it uses data from the latest Argentina Census in 2010. In fact, and in a more general sense, the main limitation of our approach is related to the availability of alternative data sources. In Argentinian Statistical System the National Census is the only source which can be disaggregated to such granular spatial resolution.

As several years have passed since the Census was completed, having access to up-to-date information is crucial to improve is effectiveness. Because of that, the Index should be re-calculated as soon as future Census data is made public. This problem affects specially the SES Index. It is possible to assume that the values and relationships between sociodemographical indicators change over time. This would make impossible to use the weights in the autoencoder of 2010 to estimate the SES index for 2020. It would be necessary to re-estimate the SES component of the index and re-calculate the weights of the autoencoder. This issue is related to data availability and not to the autoencoder itself: if a simpler model were to be used (for example, some clustering algorithm) it should be re-estimated for newer data. However, as it has been stated in above, the aim of this paper is to develop a preprocessing workflow and a estimation methodology for calculating Sanitary Vulnerability and not to develop a model valid for different time periods.

However, some attempts to link survey and census data exist. For example, [22] uses updated but spatially limited information of Child Labor Surveys to train a model using individual predictors of child labor probability. Then, this model is expanded using the same predictors on Census data obtaining prediction at a spatially disaggregated level, but trained using updated data. Therefore, it would be possible to combine newer survey data and older Census data. A similar approach could be used using health coverage data from Permanent Household Survey in Argentina. This is a future line of work in the project.

The Index could also be improved by including highly granular spatial data from additional sources such as climate and environmental metrics.

As it is, the Map has opened up two interesting further lines of work. First, Sanitary Vulnerability can be considered a traversal dimension that affects the evolution, transmission and prevalence of different pathologies. So, a future line of work could use the Sanitary Vulnerability Map as an input to study the correlation of Sanitary Vulnerability with the spread of infectious diseases or the prevalence of chronic ones. Second, the methodology can be applied to different contexts. For example, the estimation of travel distance to health facilities can be replicated for other Public services: education, security, social assistance, housing, etc. This opens up the possibility to generate highly disaggregated information that could be useful to take cost-effective decisions in the allocation of public resources. Third, the method developed in this paper would make possible to replicate this index calculation for different time periods. When results of next Census are available it would be possible to compare changes in Sanitary Vulnerability with the highest spatial resolution allowed by public data in Argentina.

## Data Availability

This study was a re-analysis of existing data, which is openly available at locations cited in the reference section. Results of re-analysis can be accessed at https://poblaciones.org/2020/09/23/vulnerabilidad-sanitaria/. The code used during the current study are available from the corresponding author on reasonable request.

## Footnotes

1 The research that sustains this paper has been funded by the Bunge & Born Foundation.

2 Access to health is more than just physical proximity. In an optimal scenario we would include a variable reperesenting the percentage of the population that benefits from or health insurance or public health coverage. The impediment -as we will discuss in the results section- is that currently available Census Data is not suitable. The question about health coverage was only asked to a sample of the population, and its results are available only at County level (a much coarser spatial unit than the Census Block). However, we use available health coverage data as part of a validation test for our index -as discussed later.

3 The term “scrapping” or “web scrapping” refers to a set of techniques and technologies used to extract information from websites when no simple data extraction mechanism -like a download link- is provided.

4 The lead reviewer was Roberto Chuit, MD., Executive Director of Institute of Epidemiological Research in Argentina’s National Academy of Medicine and former Minister of Public Health for the Cordoba Province of Argentina.

5 INMAT is the acronym for the Construction Materials Index, an official statistic. It reflects the quality of the materials with which houses are built (considering the predominant material used for floors and ceilings). It takes into account building strength, proofing, and finishing.

6 The expanded questionnaire is applied to a sample of the total population and contains occupational, health coverage and other variables. For more information, consult [5].
